# Potential Factors of Diabetes in Gitelman Syndrome and the Choices of the Appropriate Hypoglycemic Drugs: A Literature Narrative Review

**DOI:** 10.3390/cimb48020147

**Published:** 2026-01-28

**Authors:** Izabela Szubert, Aleksandra Cader-Ptak, Ewa Kwiatkowska

**Affiliations:** Department of Nephrology, Transplantology and Internal Medicine, Pomeranian Medical University, 70-111 Szczecin, Poland; izabelaszubert2312@gmail.com (I.S.); aleksandra.cader@wp.pl (A.C.-P.)

**Keywords:** Gitelman syndrome, tubulopathy, glucose metabolism, hyperglycemia

## Abstract

Gitelman syndrome (GS) is a rare, autosomal recessive salt-losing tubulopathy caused by mutations in the *SLC12A3* gene. It involves dysfunction of the sodium-chloride cotransporter positioned on the apical membranes of the distal convoluted tubule cells, causing sodium shortage and mimicking the use of thiazide diuretics. Hyperaldosteronism secondary to sodium depletion and hypovolemia causes hypokalaemia and metabolic alkalosis. This is associated with inhibition of the Transient Receptor Potential Cation Channel, Subfamily M, Member 6 –TRPM6 channel, which leads to urinary magnesium leakage and hypomagnesemia, subsequently stopping PTH secretion and resulting in hypocalcemia and hypocalciuria. Gitelman syndrome frequently presents later in life, as the symptoms are usually not very threatening. However, early identification, diagnosis, and urgent intervention are essential to improve patient prognosis and quality of life. Importantly, both hypomagnesemia and hypokalaemia can impair insulin secretion and sensitivity. Furthermore, hyperaldosteronism caused by the secondary activation of the R-A-A system can also lead to these disorders. Glucose metabolism problems have been shown to prevail amongst GS patients and manifest more frequently in comparison to the general population. When it comes to the treatment used to reduce hyperglycemia in GS-related T2DM, we consider which of the available drugs are the best for those patients. The article analyses the association of Gitelman syndrome with diabetes mellitus based on the available medical literature—as there are no clinical trials or meta-analyses available for this group, it is presented as a narrative review.

## 1. Introduction

Gitelman syndrome (GS), also referred to as familial hypokalaemia–hypomagnesemia, is a rare, autosomal recessive salt-wasting tubulopathy caused by mutations in the SLC12A3 gene encoding the thiazide-sensitive sodium-chloride cotransporter (NCC) expressed in the apical membrane of the cells lining the distal convoluted tubule. The most common mutation may be Thr60Met [1]. Evidence et al. showed that the majority of patients were compound heterozygotes for SLC12A3 mutations, while a significant group of patients carried only a single SLC12A3 mutation, presumably because of a failure to identify the second allele mutation [2]. Biallelic inactivating mutations are diagnostic features. Confirmation after suspicion of GS requires a genetic test, which should be offered to all the patients. The diagnosis of GS is proven with a genetic test that reveals the mutation. GS can be inherited or acquired. The human SLC12A3 gene is responsible for encoding the thiazide-sensitive Na-Cl cotransporter (NCC) located in the apical membrane of the distal convoluted tubule cells. The authors’ exploration of the human genome database allowed them to find 488 mutations of the SLC12A3 gene presented in patients suffering from Gitelman syndrome. Among them were, for instance, missense mutations, shear mutations, deletion mutations, nonsense mutations, reading frame shift mutations, and others [1]. The occurrence of hypokalaemia in patients aged over 55 and carrying DM varied between 1.0 and 1.2% [3]. The diagnosis is mainly based on symptoms, laboratory tests, and genetic tests. It most often presents in adolescence or early adulthood. Because of the diversity of its symptoms, early-onset GS can be initially misdiagnosed. GS patients can be asymptomatic in the first years of life, and symptoms appear periodically or are transitory. The most characteristic qualities are hypokalaemic metabolic alkalosis, hypomagnesemia, hypocalciuria, normal or lower blood pressure, paraesthesia, palpitation, and cramps at night or during moderate physical activity, which results from the electrolyte imbalances. Thirst, nocturia, and salt cravings are also common symptoms, with three-fourths of patients experiencing increased thirst and salt cravings and many instead choosing pickle brine, salted cucumbers, and citrus fruit [4]. Polyuria, ataxia, joint pain, and blackouts can also appear, though rarely. Chondrocalcinosis developing in patients with Gitelman syndrome is linked to hypomagnesemia, because Mg is a cofactor for phosphatases. Hypomagnesemia leads to an increased concentration of the pre-cursor of calcium pyrophosphate crystals—extracellular ionic inorganic pyrophosphate. Deposition of the crystals in the articular cartilage results in chondrocalcinosis [5]. In very rare cases, ventricular tachycardia, life-threatening sudden cardiac arrest, seizure disorder, rhabdomyolysis, and acute flaccid paralysis can develop as well. Case et al. discussed an 8-year-old Japanese boy experiencing a generalised convulsion that lasted for 5 min, with hypokalaemia and metabolic alkalosis. These findings prompted the authors to consider whether the patient had GS at his initial presentation. An examination of his SLC12A3 gene for mutations revealed that he was suffering from Gitelman syndrome [6]. Cardiac arrhythmia and seizures are the most serious complications resulting from these metabolic disorders. Another case showed the clinical state of a 43-year-old woman with cardiac arrest. Upon admission, her laboratory tests revealed hypokalaemia and hypomagnesemia with prolonged QTc. She was subsequently diagnosed with GS [7]. An electrocardiogram may also reveal QT prolongation. Prolongation of the QTc interval, which is a consequence of potassium and magnesium disturbances, predisposes patients to dangerous arrhythmias. This may be a consequence of hyperglycaemia. Data et al. claimed that patients with T2DM were more susceptible to QTc prolongation [8]. GS may be accidentally misdiagnosed due to its low morbidity, nonspecific symptoms, or ignorance. Renin–angiotensin system (RAS) activation is essential in the pathophysiology of GS. The loss of function in the tubular channels responsible for sodium absorption leads to volume status changes and electrolytic disturbance. The renal loss of sodium and volume depletion activate the renin–angiotensin–aldosterone system [9]. GS is typically diagnosed incidentally. The condition is characterised by its typically asymptomatic nature or the manifestation of mild symptoms, yet it is recognised to be associated with a substantial diminution in quality of life. Patients with GS are traditionally treated with oral potassium and magnesium supplementation. Rujie et al. suggested using potassium-sparing diuretics in the event of continued symptomatic hypokalaemia, insufficient chronic supplementation or unbearable side effects [10]. Renin–angiotensin system blockers can be used as well, as they halt the activated renin–angiotensin–aldosterone system. In the present paper, the available medical literature is analysed in order to explain potential pathomechanisms that can be conductive to the prevalence of these ion pathologies and their clinical consequences.

## 2. Hyperglycaemia

Diabetes mellitus is divided into two main types: insulin-dependent diabetes mellitus—type 1, and type 2 diabetes mellitus. The former is as an autoimmune disorder. The main pathologies are related to T lymphocytes and their reactivity against pancreatic β-cells. T2DM, in turn, is a chronic disease characterised by hyperglycaemia resulting from improper insulin secretion or insulin function, or both. Maintaining hyperglycaemia over a prolonged period is the main risk factor for heart infarction, stroke, kidney disease, retinopathy, and extremity amputation.

A group of glucose transport proteins expressed in specific tissues referred to as GLUTs redounds to the glucose uptake process. In the adipose tissue and skeletal muscles, GLUT4 is the main isoform. IR and type 2 diabetes mellitus are associated with impaired GLUT4 contents in skeletal muscles and the adipose tissue. As a result of such impairment, the system increases serum insulin concentration as a compensatory mechanism. Depending on how advanced the process is, it can easily lead to developing diabetes mellitus. This disease involves pancreatic dysfunction in insulin exocytosis, accompanied by insulin resistance [11].

**Figure 1:**
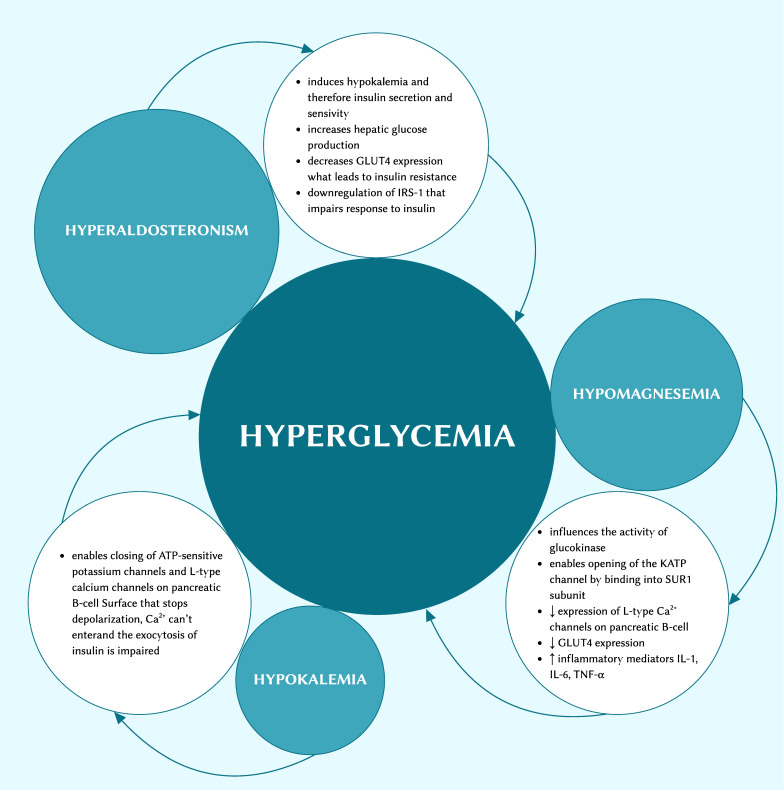
The linkage between ion disorders and hyperglycaemia.

GS patients suffer from hypokalaemia, hypomagnesemia, and hyperaldosteronism, with each potentially leading to glucose intolerance (Figure 1). In their research, Hong Ren proved that compared with a healthy normal control group, GS patients showed a higher area under the curve (AUC) glucose level and AUC insulin level [12]. This indicates an increased probability of hyperglycaemia and insulin resistance [12].

In Chinese GS patients, 14 to 60% may have abnormal glucose metabolism. Tao Yuan et al. showed that the patients with Gitelman syndrome clearly had higher glucose levels than healthy patients did at 60, 120, and 180 min in OGTTs [13]. Both GS and DM patients showed similar trends in glucose and insulin levels, with a glucose peak at 60 min [13]. Insulin resistance (IR) was a common clinical condition among these patients. IR is a state of decreased influence of insulin on different tissues where the serum concentration of insulin is normal or even where it is increased. IR is a chronic and pathological condition that is related to reduced glucose uptake, especially in target tissues, including liver, muscles, and the adipose tissue, and occurs when insulin receptors lose their sensitivity to insulin. It is the most important feature of type 2 diabetes mellitus. Ren et al. proved that excessive aldosterone aggravated the rate of glucose uptake through GLUT2 and GLUT4. The translocation of GLUT4 to the plasma membrane was also impaired [12].

Davis et al. discussed cases of 6 patients with BS/GS (1 with BS, 5 with GS) and 10 normotensive healthy individuals having oral glucose tolerance tests [14]. All had normal oral glucose tolerance tests at baseline and at 120 min. Insulin at baseline was slightly decreased compared with control subjects, while there was no difference at 120 min. Oral glucose insulin sensitivity was higher in BS/GS patients. These results pointed to alleviated insulin resistance in BS/GS patients [14].

Guangyu He et al., in their case report, described the clinical history of a 55-year-old Chinese male patient with intermittent fatigue, occasional limb weakness, stiffness, and palpitations [15]. Testing blood electrolyte levels showed hypokalaemia, hypomagnesemia, and mild hypochloraemia. Urine analysis revealed kaliuresis, natriuresis, chlorosis, and hypocalciuria. These symptoms were aggravated upon an intake of juice. The patient was subsequently diagnosed with type 2 diabetes mellitus. The pathogenic mutation for GS was found in the SLC12A3 gene [15].

Also, Xiaoyan Huang et al., in their case report, described the clinical condition of a 36-year-old male patient diagnosed with diabetes mellitus 14 years prior [16]. The patient was managed with oral hypoglycaemic agents and had a longstanding history of hypokalaemia, which was revealed 15 years earlier. Genetic tests showed that the condition was a symptom of GS. A co-occurrence of diabetes mellitus and Bartter syndrome was also noticed in his sister [16].

## 3. Hyperaldosteronism

Aldosterone is a steroid hormone and the final mediator of the renin–angiotensin–aldosterone system. It is responsible for regulating the water–mineral balance and mediating blood pressure; it induces vascular dysfunction and remodelling. Moreover, aldosterone boosts NAD(P)H oxidase activity and reactive oxygen species (ROS) generation [17].

An increased NaCl loss causing hypovolemia due to secondary water loss activates the R-A-A system. RAAS has been well-known to play a core role in the regulation of extracellular volume, sodium, and potassium plasma concentration. Firstly, thanks to the function of the macula densa cells, renin is produced. This compound is necessary to transform angiotensinogen into angiotensin I. Subsequently, thanks to the angiotensin-converting enzyme, which is ubiquitously released from endothelial cells or others, angiotensin I transforms into angiotensin II. The action of angiotensin II at the AT_1_R leads to enhanced sodium retention, vasoconstriction (including constriction of the efferent arteriole of the kidney), escalated thirst and salt craving, boosted sympathetic nervous system activity, and the releasing of the aldosterone from the adrenal gland’s zona glomerulosa [18].

The pathogenesis of glucose metabolism abnormalities in patients with PA appears to be multifactorial: the effects may be mediated by the influence of hypokalaemia on insulin sensitivity and insulin secretion, and by aldosterone’s action on the pancreatic β-cells or on insulin resistance. In their article, Jeon et al. highlighted that the progression of the growth of plasma aldosterone and BMI was supported by recent evidence that the novel adipokine complement-C1q tumour necrosis factor-related protein 1 (C1q-TNF) chronically stimulated the secretion of aldosterone and was elevated in the adipose tissue in rodents with obesity [8]. CTRP1 expression is brought about by proinflammatory cytokines that can appear in large part as a consequence of hypomagnesemia [19].

Clinical reports suggest that patients with hyperaldosteronism more frequently demonstrate impaired glucose tolerance, but the exact mechanism is not yet fully clear. RAAS activation leads to an elevated content of the main vasoconstrictor peptide—angiotensin II (Ang II). Ang II has an impact on glucose homeostasis and is involved in DM pathogenesis through decreasing insulin signal transduction, reducing glucose uptake, insulin resistance, and destroying the β-cells of the pancreas by generating oxidative stress [20]. Furthermore, mineralocorticoid receptors have a high affinity for glucocorticoids. Aldosterone also activates the glucocorticoid synthesis, and by this influence on glucose metabolism, it leads to inflammation and insulin resistance. Furthermore, aldosterone may increase hepatic glucose production via elevating glucose-6-phosphatase, fructose-1,6-bisphosphatase, and phosphoenolpyruvate carboxykinase levels. Aldosterone may also change insulin sensitivity by impairing normal adipocyte functions, such as differentiation and function. In vitro pre-adipocyte differentiation via the mineralocorticoid receptor is induced by aldosterone. Adiponectin is the best-characterised adipose-derived circulating hormone, or adipokine, thanks to which insulin sensitivity is improved. Aldosterone excess is also connotated with reduced circulating adiponectin and visceral adipose adiponectin expression [21].

Aldosterone increases insulin resistance in isolated vascular smooth muscle cells (VSMC) by reduced IRS1 expression and decreased Akt signalling. Aldosterone works via MR and Src-kinase activation, and the expression of VSMC insulin-like growth factor-1 receptor (IGF1R) is increased. A hybrid IR/IGF1R receptor can be formed, and then further MAP-kinase pathway activation promotes vascular hypertrophy. In consideration of the aforementioned mechanisms, it is recognised that excess aldosterone levels have a regulatory impact on insulin resistance, sensitivity, and β-cell function.

In Fischer et al.’s [22] study of β-cell function, ivGTT and arginine challenge tests were performed. First-phase insulin release (FPIR) in response to glucose was significantly reduced in patients with primary hyperaldosteronism in comparison to normal controls. The authors also observed a trend toward a lower FPIR level during ivGTT in primary hyperaldosteronism compared to healthy controls. A significant effect of adrenalectomy on early insulin secretion was found in these patients.

In addition to the aforementioned data, studies indicate the potential for hyperaldosteronism to impact GLUT receptor function. Jayaraman et al., in their study of healthy Wistar strain adult male albino rats, revealed that extra aldosterone impaired the glucose uptake rate through a faulty expression of GLUT2 and GLUT4 genes and also reduced GLUT4 translocation to the plasma membrane, which influenced insulin resistance [3].

## 4. Hypokalaemia

Potassium is a crucial micronutrient in the human system. It is the first-most abundant intracellular cation, with approx. 98% of the body’s K located in intracellular fluid. The element is responsible for maintaining the electrical action potential across cell membranes, regulating cell metabolism, glycogen, and cardiovascular functioning. Low K^+^ concentrations can delay the cardiac muscle’s repolarisation, which may contribute to both atrial and ventricular arrhythmias. ECG changes that appear regularly are T wave flattening, ST-T segment depression, QT interval prolongation, and presence of U waves. Different ventricular extrasystoles can be observed in up to 20% of patients with severe hypokalaemia (>2.6 mmol/L) [23]. Hypokalaemia in adults can be a common problem. Hypokalaemia, by definition, is a plasmatic potassium concentration of <3.5 mEq/L. In the event of an identification of potential risk factors for hypokalaemia, the following medications should be taken into consideration: insulin, beta-2 agonists, antiarrhythmic agents, glucocorticoids and mineralocorticoids, antibiotics, antifungals, and laxative overdosing. There is no specific pattern of symptoms, which often depend on intrinsic factors and the clinical status of the individual patient. Hypokalaemia can lead to worse consequences in diabetic patients, as they are at a greater risk of developing chronic hypokalaemia. In this group of patients, cardiovascular diseases are frequent, which makes them more susceptible to cardiac arrhythmias, fluid depletion, and neuropathy aggravation [23]. Mild hypokalaemia can be asymptomatic and overlooked, but in Coregliano-Ring et al.’s study, in patients suffering from comorbidities, moderate hypokalaemia was demonstrated to increase the risk of morbidity and mortality when compared with healthy control individuals [3].

Pancreatic ATP-sensitive potassium channels play a crucial role in coupling glucose metabolism to the secretion of insulin in response to an increase in ATP concentration. The channel should be considered as a key therapeutic milestone in finding the best treatment for diabetes mellitus. As soon as the intracellular ATP/ADP ratio increases, KATP channels close, and the result is membrane depolarisation. This in turn promotes the plasma membrane’s voltage-gated L-type Ca^2+^ channels to open, following which Ca^2+^ enters, and the exocytosis of insulin-secreting granules is stimulated. Hypokalaemia can enable the closing of ATP-sensitive potassium channels and L-type calcium channels on the pancreatic β-cell surface, which prevents an increase in calcium concentration in the cell and stops insulin exocytosis.

At the end of the study performed by Chatterjee et al. [24] participants taking potassium supplements showed markedly better fasting glucose levels compared to those taking a placebo. A total of 8 of the 15 participants taking KCl experienced improvements in fasting glucose levels ranging from −1 to −19 mg/dL. Amongst the participants taking a placebo, 2 out of 12 had improvements in fasting glucose contents.

Oana C Iatcu scanned mineral intake as a percentage of the recommended dietary intake and observed that all patients, both those with diabetes mellitus and prediabetic ones, had a statistically significant reduction in potassium intake in comparison with the RDI [25].

Natural et al. [25] in a prospective cross-sectional study, suggested an inverse relationship between the dietary intake of minerals and body fat. Individuals with obesity had lower consumption of potassium compared with overweight individuals and individuals with normal weight. These relations undoubtedly show how low potassium intake can lead to weight gain and trigger insulin resistance as an element of diabetes mellitus.

Tariq Shafi et al. [26], during year 1 of their research into the chlorthalidone-induced changes in serum potassium, showed that a greater decrease in serum potassium was associated with a higher unadjusted IR of diabetes mellitus than in the placebo group. Each 0.5 mEq/L decline in serum potassium content from the average baseline level entailed a 45% higher risk of incident diabetes mellitus from the beginning to the end of the study period [27].

## 5. Hypomagnesemia

Magnesium is another of the most essential micronutrients in humans, being the second-most abundant intracellular cation after K^+^. As a cofactor for numerous energy-metabolising enzymes like hexokinase, creatine kinase, and protein kinase, but also as a component of the Mg^2+^-ATP complex, it takes part in different physiological and pathological processes. Magnesium is a cofactor for more than 600 enzymes and activates over 200 enzymes. Mg^2+^ can bind inorganic phosphate, ATP, phosphocreatine, and others. It has a significant impact on many metabolic reactions, especially those connected with carbohydrate metabolism and cellular bioenergetics [28]. It has been recognised as a key factor in reducing oxidative stress damage. It may also promote inflammation in the human organism. The reabsorption of Mg^2+^ in the ductal convoluted tube (DCT) depends on the proper functioning of a sodium-chloride cotransporter, the NCC. Moreover, recent studies have explored the role played by calcineurin and uromodulin (UMOD) as regulators of both NCC and Mg^2+^ handling by the DCT. Calcineurin inhibitors lead to hypomagnesemia in a state of NCC activation, which can also be a consequence of direct effects on TRPM6 gene expression. In Umod^−/−^ mice, the presence of hypomagnesemia may be partly due to a lower NCC expression and a decreased TRPM6 expression on the cell surface [29].

It is worth mentioning that intracellular Mg^2+^ balancing is crucial for proper carbohydrate metabolism. Low intracellular concentrations of Mg^2+^ and/or thiamine diphosphate (TDP) are risk factors that can switch the oxidative metabolism of glucose [30]. Magnesium deficiency contributes to DM. Magnesium content drops have also been associated with DM in several cohort studies, and magnesium supplementation in diabetics is associated with a decrease in fasting glucose levels. For example, in Paolisso et al.’s [30] research, their results showed that chronic magnesium supplementation had a real effect on insulin response and action, and both appeared to improve significantly after chronic magnesium administration. They observed an increase in acute insulin response to glucose pulse, and an increase in the glucose infusion rate calculated in the last 60 min of a euglycemic–hyperinsulinemic glucose clamp [31]. 

A mounting quantity of evidence suggests a correlation between elevated Mg^2+^ serum levels and enhanced insulin sensitivity, along with a reduced likelihood of developing type 2 diabetes mellitus (T2DM). Yiqing Song et al., in their meta-analysis of the effects of oral magnesium supplementation, pointed out that daily supplementation of 250 mg of Mg^2+^ in T2DM patients for 4–16 weeks might be effective in reducing plasma fasting glucose levels [32].

In their research, A. Hruby et al. [33] showed that, compared with those with the lowest magnesium intake, patients receiving the highest dosage had a 37% lower risk of incident metabolic impairment, while in those with baseline metabolic impairment, a higher intake was associated with a 32% lower risk of incident diabetes mellitus. In the combined population, the risk in those with the highest intake was 53% of that demonstrated by subjects with the lowest intake. A higher magnesium intake tended to be associated with lower follow-up FG and IR, but not fasting insulin, postload values, or insulin sensitivity.

The effect of magnesium supplementation on fasting blood glucose levels has been demonstrated in several studies. Veronese et al. [34], in their meta-analysis, proved that Mg supplementation significantly reduced fasting plasma glucose at follow-up in 325 participants with diabetes mellitus compared to 331 individuals receiving placebo. Furthermore, they demonstrated that individuals with a high probability of developing diabetes mellitus who received magnesium (Mg) supplementation experienced a substantial reduction in plasma glucose levels, as evidenced by both a 2 h oral OGTT and a per se evaluation. In addition, Mg supplementation improved insulin sensitivity markers.

The normal reference range for serum Mg^2+^ content is 0.76–1.15 mmol/L. Magnesium deficiency occurs when serum Mg^2+^ concentration is ≤0.75 mmol/L (1.8 mg/dL) or >2 SD below the mean value for the general population. At that point, preclinical hypomagnesemia can be taken into consideration. When serum Mg^2+^ concentrations decrease to ≤0.61 mmol/L (1.5 mg/dL), frank hypomagnesemia can be recognised. Magnesium deficiency (MgD) can present without hypomagnesemia in laboratory tests. Intracellular free Mg^2+^ levels are reduced in patients with T2DM, in comparison to nondiabetic patients. There is a great deal of symptoms that can result from hypomagnesemia, among them anxiety, irritability, headache, muscle spasm, depression, poor memory, paraesthesia, disorders of vitamin D metabolism, arrhythmias, hypertension, endothelial dysfunction, increased platelet aggregation, sodium retention, hypokalaemia, and hypocalcaemia. There are several clinical manifestations of hypomagnesemia that may include tremors, fasciculation, tetany, and convulsions. Neuropsychiatric disorders can also develop, such as apathy, delirium, and even comas [35].

Magnesium plays a role as a cofactor in adenosine triphosphate- and tyrosine kinase-dependent reactions that are essential to glucose metabolism in β-cells in the pancreas. As the first step in insulin secretion, glucose uptake into the pancreatic β-cell is mediated by GLUT2. In the ATP production pathway in β-cells, glucose is converted to glucose-6-phosphate by glucokinase. The activity of glucokinase depends on magnesium-ATP. The tricarboxylic acid cycle and oxidative phosphorylation are glucose metabolic pathways. Numerous enzymes in these metabolic pathways require magnesium. The insulin receptor is composed of two alpha and two beta subunits. In the insulin signalling process, insulin binds to the alpha subunit, following which the tyrosine kinase (TK) in the beta subunit is activated. The opening of the KATP channel depends on the binding of Mg-ATP to the SUR1 subunits. The Mg-ATP complex is an important element in glycolytic enzymes and in phosphorylation reactions. A suboptimal magnesium level reduces glucokinase activity and glucose binding to glucokinase, hindering insulin release, which then results in insulin deficiency. The GLUT2 and glucokinase (GK) tandem is often referred to as a glucose sensor controlling blood sugar levels. Mechanisms altering the glucose-dependent activity of GK effectively adjust the set point for whole-body glucose homeostasis. A lack of magnesium may impair the functioning of the GK, glucose-6-phosphatase G6P formation, and the accumulation of ATP in β-cells, which may enable the closing of the KATP channels. This delays early and late plasma insulin responses to glucose. While ATP binds to Kir6.2 subunits of the pancreatic β-cells, the KATP channel closes. Mg-ATP and Mg-ADP bind to SUR1, which brings about the reverse result of opening the channel. When KATP channels close, the β-cell membrane depolarises, which stimulates Ca^2+^ influx thanks to L-type Ca^2+^ channels, and insulin is unleashed. It is also possible that a reduced amount of magnesium decreases the expression of L-type Ca^2+^ channels, which indirectly reduces insulin secretion. KATP channel closure and the depolarisation of the β-cell membrane promotes the influx of Ca^2+^ through the L-type Ca^2+^ channels as well as insulin release. This phenomenon can commence when the potential of the membrane reaches approximately −50 mV [36].

Recent research also suggests that Mg^2+^ is a pivotal factor in the functioning of liver enzymes. For instance, it plays crucial roles in gluconeogenesis and glycogenesis pathways in the liver.

Inflammation is known to be a very important factor in developing insulin resistance. A meta-analysis revealed that Mg supplementation significantly reduced serum C-reactive protein (CRP) and escalated nitric oxide (NO) levels. What is worth mentioning is that magnesium taken as a supplement reduced plasma fibrinogen, tartrate-resistant acid phosphatase type 5, tumour necrosis factor-ligand superfamily member 13B, ST2 protein, and IL-1 levels. In conclusion, thanks to Mg supplementation, the levels of different human inflammatory markers such as CRP may be reduced [37].

Oxidative stress is another key state promoting T2DM. Persistent chronic hyperglycaemia can lead to this clinical condition. One review described how magnesium deficiency enhanced the recruitment of phagocytic cells to perform their effector functions, which caused the generation of reactive oxygen species (ROS). Prolonged ROS production can have a pathological effect on tissues, which is why inflammatory stress is assumed to be a risk factor for several chronic diseases [38].

Various studies have demonstrated that magnesium levels can also have a bearing on the occurrence of diabetic complications. The results presented by Zhang Yiyan et al. showed that lower serum magnesium—less than 3.5 mmol/L—was the associated factor in male and female diabetic patients suffering from common diabetic complications, such as diabetic retinopathy and diabetic nephropathy [39].

In their study, Weichao Huang showed that patients who underwent an analysis upon a high magnesium intake (i.e., a dosage of 267 mg per day or more) had lower 2 h glucose (2hOGTT) values and a lower BMI [34].

What is more, Mg may have an important effect on factors having an impact on glucose metabolism, such as body composition, general health, and sleep quality. ELDerawi et al. explained that a daily intake of 250 mg of Mg^2+^ in a group of T2DM patients improved their HbA1c, insulin levels (ILs), C-peptide, and HOMA-IR, and subsequently reduced their IR. Moreover, after 3 months of the intervention, their glycaemic control indicators improved [40].

Guerrero-Romero et al. [41] described complicity with Mg^2+^ levels, inflammation, and oxidative stress as risk factors for the development of metabolic syndrome (MetS). Analysis was conducted over a 10-year horizon. As compared with the placebo, 22.3% and 22.0% of men supplementing MgCl_2_ did not develop diabetes mellitus. The authors concluded that using oral MgCl_2_ for at least 4 months, in adults with prediabetes and hypomagnesemia, was a profitable possibility or one way of reducing the prevalence of complications and the direct medical costs.

## 6. DM Treatment in Gitelman Syndrome

As outlined in the preceding case study, this patient exhibited hypoglycaemia recurrence after the administration of potassium and magnesium supplementation therapy. We explored the possible reasons for this case of hypoglycaemia and emphasised the importance selecting suitable hypoglycaemic drugs in such patients.

**Figure 2:**
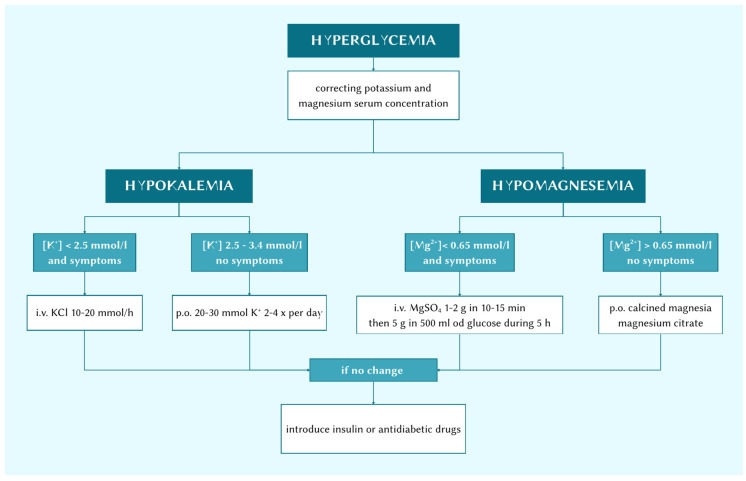
Treatment options.

The first step of the treatment should be to administer potassium and magnesium supplements. These elements are known to have a crucial impact on glucose metabolism and thus can sometimes be enough to remove chronic hyperglycaemia (Figure 2).

Insulin is an obvious alternative for hypoglycaemia treatment. In the event that β-cells are unable to secrete insulin, the option of administrating the hormone to the patient is available. Clearly, in the event of insulin resistance, there is no certainty that this will bring about the expected results. It is, however, the first, and at the same time, safest option to deal with decreasing blood glucose levels, as initially careful doses can be applied and modified quickly if the dosage is inappropriate. After insulin administration, the sodium–potassium ATPase pump is activated, and K^+^ enters the peripheral cells. At average levels, insulin induces only a transient reduction in serum K^+^ concentrations, which will not cause any significant changes in potassium levels. With high doses of insulin, hypokalaemia can sometimes be exacerbated as a side effect. Caution must be exercised to avoid high-dose insulin administration. Insulin use when blood potassium levels fall below 3.3 mmol/L may elicit severe arrhythmias and respiratory muscle weakness as it aggravates hypokalaemia as a consequence of potassium entering the cells.

Dipeptidyl peptidase-4 (DPP-4) inhibitors or glucagon-like peptide-1 (GLP-1) are considered to be appropriate drugs for use in GS patients with DM. Gut-derived glucagon-like peptide-1 (GLP-1) belongs to the family of incretin hormones secreted by enteroendocrine L cells of the small intestine. Its analogues not only increase the exocytosis of insulin by stimulating the production of GLP-1 by L cells in the small intestine without influencing the receptor but also slow down gastric emptying and, as a consequence of the latter, reduce and/or delay glycaemic increases after meals. They also cause the suppression of glucagon secretion during hyper- or euglycaemia and additional weight reduction.

DPP4 inhibitors known as gliptins are oral antihyperglycemic agents employed in the treatment of type 2 diabetes mellitus, with abundant clinical experience gathered since their approval. They are responsible for slowing the inactivation and degradation of GLP-1. Gut–derived incretins increase insulin secretion and suppress glucagon release in response to glucose supply. They are generally well–tolerated, weight–neutral, demonstrate intermediate effectiveness in reducing HbA1c levels, and do not increase the risk of hypoglycaemia. They can be utilised regardless of the patients’ comorbidities, such as atherosclerotic cardiovascular disease (ASCVD), heart failure (HF), or chronic kidney disease (CKD). They do not only have antihyperglycaemic effects, as they also demonstrate antihypertensive, anti-inflammatory, antiapoptotic, and immunomodulatory impacts on the heart, kidneys, and blood vessels, regardless of the incretin pathway. Their next advantage is that they very rarely cause hypoglycaemia. There have been reported cases of acute pancreatitis as a side effect, which is why patients carrying a higher risk of developing this condition should avoid this group of drugs. A meta–analysis of their clinical trials showed that taking DPP-4 inhibitors was associated with a 44% risk of causing acute pancreatitis, while GLP-1 administration was correlated with a risk of 60% in comparison to placebo/active comparator [42].

Sodium–glucose cotransporter 2 (SGLT2) inhibitors are a novel class of antihyperglycemic drugs recently approved for diabetes mellitus treatment. Nowadays, they are very popular and valued due to their positive impact on the cardiovascular system. This is why they have been approved for use in patients with chronic kidney disease or heart failure aiming to reduce the risk of heart attack and stroke, including in individuals without diabetes mellitus. Na^+^ delivery to the macula densa is increased, and thus, SGLT2-inhibitors promote the vasoconstriction of the afferent renal arterioles and the reduction in hyperfiltration-mediated inflammatory pathways. Influencing renal function and/or mitigating renal stress are plausible actions that can slow down the progression of HF through multiple pathways. Some worth mentioning are the decreasing of afferent sympathetic nervous system activation, inflammation reduction, and ROS production inhibition [43].

SGLT2 is a major transport protein responsible for reabsorption from the glomerular filtration of glucose back into circulation, which covers almost 90% of the kidney’s glucose reabsorption. The drugs discussed here inhibit renal glucose reabsorption in the early proximal tubule, thus causing glucosuria and lowering the glucose burden on the system. SGLT2 inhibitors do not increase the risk of hypoglycaemia because they stop lowering blood glucose levels once the filtered glucose load falls to ~80 g/day, although they can exacerbate volume drop and hypotension. They may be used to control the blood glucose level and increase the blood concentration of magnesium. They increase the PTH level, stimulate Mg^2+^ reabsorption in the thick limb, and upregulate TRPM6/7 in DCT. They can also be beneficial in Mg^2+^-wasting disorders [44].

The glucose-lowering potential of SGLT-2 inhibitors becomes compromised with a progressive decline in kidney function. SGLT-2 inhibition can result in sodium reabsorption suppression and the occurrence of glucosuria, both of which have the potential to exacerbate hypovolemia. Enhanced osmotic diuresis after the introduction of SGLT2i is associated with a decrease in the estimated plasma volume. SGLT2i promotes natriuresis; therefore, a 7% plasma volume reduction is visible. This contributes to BP lowering by an average of approx. 4/2 mm Hg [44].

Moreover, Taha Ahmed et al. reported a case of euglycemic diabetic ketoacidosis in a GS patient who had been using SGLT2i. This phenomenon is a scarce but not uncommon side effect of this drug. A 51-year-old female presented with recurrent, diffuse, continuous and sharp stomach-ache radiating to her back and accompanied by nausea. She struggled with chronic hypokalaemia and hypomagnesemia, and type 2 diabetes mellitus (at the moment being treated with metformin, pioglitazone, and empagliflozin—the last one for three months). In an arterial blood gas analysis, there was an anion gap metabolic acidosis, severe hypokalaemia, and acute kidney injury. Elevated ketones were both in the blood and urine. All tests allowed for a suspicion of euglycemic DKA secondary to empagliflozin [16].

Xiaoyan Huang et al. [16], in their literature review, suggested using α-glucosidase inhibitors in order to delay after-lunch carbohydrate absorption and moderate postprandial hyperglycaemia. Their use is not associated with a substantial risk of hypoglycaemia.

Metformin is a synthetic biguanide, and an orally effective and insulin sensitising antidiabetic drug. It is almost always administered to most patients as a first-line antihyperglycaemic for the treatment of type 2 diabetes mellitus. It attenuates the ability of glucagon or inhibits mitochondrial GPD (glycerol-3-phosphate dehydrogenase), subsequently leading to an impairment of lactate utilisation for gluconeogenesis. Studies suggest that metformin could also enhance GLUT1 (glucose transporter 1), mediating glucose transport into hepatocytes through activating IRS2 (insulin receptor substrate 2), which leads to a decrease in plasma glucose levels. Recently, it has been proven that metformin directly targets FBP1 (fructose-1,6-bisphosphatase-1). One side effect of using metformin may be lactic acidosis, especially when hypovolemia is present. MALA develops when lactates are accumulated due to a reduced clearance by oxidative phosphorylation in the mitochondria. Oxidative phosphorylation is induced by metformin and leads to mitochondrial dysfunction, and subsequently to lactic acidosis, even under the conditions of normal oxygen supply [45].

Cizmecioglu et al. [46] described a case of a 47-year-old male GS patient experiencing weight loss and suffering from xserostomia. Laboratory tests revealed an HbA1c level of 11.8%. The patient received metformin + gliclazide-based treatment accompanied by oral potassium and magnesium supplementation. After the first month, his blood potassium level improved, and his hyperglycaemia was normalised. This demonstrated the frequent co-occurrence of DM and GS, and another possible treatment method.

Sulfonylureas are drugs that have had the longest history of use. Over the past half-century, they have been administered as the main oral antidiabetic drugs to promote insulin secretion in patients. They exert their hypoglycaemic effect by stimulating insulin secretion in a glucose-independent manner, through an interaction with specific receptors located on the surface of pancreatic β-cells. The therapeutic target of sulfonylurea drugs consists of antagonising Mg-ATP binding to SUR1. This enables channel opening and induces channel closure. In MgD, the intracellular levels of ATP and MgATP decrease, which inhibits the closure and opening of KATP channels. This results in a disruption of the normal phases of insulin release. It has been demonstrated that the administration of this treatment is associated with an increased risk of weight gain and episodes of hypoglycaemia.

To summarise the above, insulin, DPP-4 inhibitors, and glucagon-like peptide-1 (GLP-1) receptor agonists are supposedly the best ones for them, but others, such as assulfonylureas, glinides, SGLT2 inhibitors, and metformin, should be avoided. Metformin can lead to lactic acidosis. SGLT-2 inhibitors can exacerbate hypotension. Serious adverse events and severe hypoglycaemia are not as common with SGLT2 inhibitors and GLP1 agonists as they are with sulfonylureas or insulin. Therefore, it is so crucial to choose appropriate hypoglycaemic drugs for GS patients, particularly when adjusting hypokalaemia and hypomagnesemia treatment.

It is crucial to emphasise that there is a paucity of clinical trials concerning hypoglycemic treatment for patients with Gitelman syndrome (GS) in the available literature. Consequently, our chapter on hypoglycemic management offers recommendations primarily based on articles detailing how ionic and endocrine disturbances (such as hyperaldosteronism) affect insulin secretion, peripheral insulin action, and overall carbohydrate metabolism. A significant portion of these articles addressed ionic imbalances and carbohydrate metabolism generally, rather than specifically within the context of GS.

## 7. Conclusions

Patients with Gitelman syndrome are more likely to suffer from glucose metabolism impairments as well. Initial factors causing diabetes mellitus in GS are hypokalaemia, hypomagnesemia, and hyperaldosteronism. All three influence insulin secretion and sensitivity by causing difficulties, although in different manners. However, there is still a lack of evidence on the specific mechanism involved in impairing insulin exocytosis and tissue sensitivity. When it comes to the treatment of DM in patients with GS, at the very beginning of the process, their hypokalaemia and hypomagnesemia should be corrected by introducing intravenous or oral supplementation, depending on the clinical condition of the patient. This paper discusses which substances are appropriate for such patients. In summary, it is hypothesised that insulin, dipeptidyl peptidase-4 (DPP-4) inhibitors, and glucagon–like peptide-1 (GLP-1) receptor agonists are the most efficacious drugs. Nevertheless, this being an intricate subject, a more in-depth investigation is required. In brief, any treatment for DM can be selected in GS patients with DM by performing frequent electrolyte monitoring. The choice of hypoglycaemic agents for GS patients requires a personalised approach after considering their comorbidities and circumstances.

## 8. Limitations

A significant limitation of this work is the very small number of articles specifically addressing Gitelman syndrome (GS) and carbohydrate disorders. This scarcity of dedicated literature precluded a structured literature review, thus necessitating a narrative approach. Many articles concerning the pathophysiology of ionic imbalances and diabetes, while relevant, were not primarily written in the context of GS. Nevertheless, in our endeavour to explain the mechanisms behind the increased prevalence of carbohydrate disturbances in GS, we leveraged these broader findings to elucidate the underlying phenomena.

## Data Availability

The original contributions presented in this study are included in the article. Further inquiries can be directed to the corresponding author.
